# Correlations Between Iron Status and Body Composition in Patients With Type 2 Diabetes Mellitus

**DOI:** 10.3389/fnut.2022.911860

**Published:** 2022-07-13

**Authors:** Chen Zimiao, Luo Dongdong, Chen Shuoping, Zhou Peng, Zheng Fan, Chen Rujun, Gong Xiaohua

**Affiliations:** ^1^Department of Endocrinology and Metabolism, The First Affiliated Hospital of Wenzhou Medical University, Wenzhou, China; ^2^Department of Endocrinology and Metabolism, The Second Affiliated Hospital of Dalian Medical University, Dalian Medical University, Dalian, China; ^3^Central Hospital of Qiaoxia Town, Wenzhou, China; ^4^College of Psychiatry, Wenzhou Medical University, Wenzhou, China; ^5^Department of Burn, 906 Hospital of the Joint Logistics Team, PLA, Wenzhou, China

**Keywords:** type 2 diabetes mellitus, body composition, visceral fat mass, android-to-gynoid fat ratio, iron metabolism

## Abstract

**Background:**

Our study aimed to investigate the association between iron metabolism and body composition in patients with type 2 diabetes mellitus (T2DM).

**Methods:**

A total of 824 patients with T2DM were enrolled. Measurements of body composition were obtained by dual-energy X-ray absorptiometry. Patients were stratified into three groups according to their sex-specific ferritin levels. Basic information, laboratory results, and body composition were collected.

**Results:**

Serum iron and transferrin saturation (TSAT) were increased significantly with increased serum ferritin (all *p* < 0.05). Total iron-binding capacity (TIBC) was decreased significantly with increased serum ferritin (*p* < 0.05). Visceral fat mass (VF), android fat/total body fat mass, android-to-gynoid fat ratio (A/G ratio), and high-sensitivity C-reactive protein were all increased significantly with increased serum ferritin (all *p* < 0.05). Patients with a high A/G ratio (A/G ratio ≧ 1) had significantly higher serum iron, ferritin, and TSAT, but significantly lower TIBC. In the model adjusted for age and gender, higher ferritin levels were associated with a higher VF (all *p* < 0.05). Serum iron was positively correlated with the occurrence of a high A/G ratio (A/G ratio ≧ 1) after the adjustment of confounding factors [an odds ratio (OR = 1.09, 95% CI, 1.02–1.19, *p* = 0.02)]. With receiver operating curve analysis, the cutoff value of serum iron for a high A/G ratio was 18.56, and the area under the curve was 0.771 (sensitivity 88.9%and specificity 63.9%, *p* = 0.01).

**Conclusion:**

Higher serum iron and ferritin concentrations were positively associated with a higher VF. Higher serum iron concentrations were positively correlated with a high A/G ratio. This study indicates the potential relationship between iron overload and the body composition in patients with T2DM.

## Introduction

Iron is an essential trace element to sustain all forms of life. It involves oxygen transport, mitochondrial respiration, and DNA synthesis (1). Furthermore, some studies have reported that abnormal iron metabolism has a role in diverse disease states. Iron deficiency causes symptoms both in the presence and the absence of anemia (2). Rossler’s study has shown that iron deficiency is also consistently associated with increased mortality in patients undergoing cardiac surgery (3). Existing research indicates iron deficiency is one of the comorbidities associated with obesity by impaired iron absorption and lower iron stores despite adequate dietary iron intake (4–6).

However, iron overload is a causative factor for non-alcoholic fatty liver disease (NAFLD) and neurodegeneration. Iron overload is present in 34.5–51.5% of patients with NAFLD (7, 8). Over the years, iron is observed to accumulate in specific brain regions during normal aging and is associated with neurodegenerative disorders (9, 10). Tambasco et al. have explored the relationship between brain iron distribution and clinical features in patients with Parkinson’s disease, evaluated by magnetic resonance imaging (11). Their findings have suggested that iron overload correlated with the majority of motor and cognitive parameters. The KORA F4 study has found that high ferritin and transferrin levels and low soluble transferrin receptors are independently related to impaired glucose metabolism (IGM) and type 2 diabetes mellitus (T2DM; 12). While increased ferritin and decreased soluble transferrin receptor probably reflect increased iron stores, body iron stores might contribute to the pathogenesis of IGM and T2DM. Iron overload may lead to cellular oxidative damage and initiate insulin secretory dysfunction in pancreatic β-cells (13, 14). Jahng et al. have described a mechanistic link between chronic iron overload and autophagy dysfunction, which altered insulin sensitivity in skeletal muscle *in vitro* and *in vivo* models (15). Additionally, iron overload is relevant to diabetic complications. Vinchi et al. have compared with normo-ferremic apolipoprotein-E (ApoE)−/−mice and found that atherosclerosis is profoundly aggravated in iron-loaded ApoE−/−FPN*^wt/C^*^326^*^S^* mice (16). Iron is heavily deposited in the arterial media layer, which is correlated with plaque formation, vascular oxidative stress, and dysfunction. Iron overload is also found in the kidney of diabetic nephropathy (17, 18). Zou et al. have shown that the oxidative stress, inflammation, and collagenous fibrosis in diabetic nephropathy rats were alleviated by iron chelating treatment (19). However, in a cross-sectional study, lower levels of serum iron were related to diabetic retinopathy (20). Therefore, there is a very complex inter-relationship between iron status, diabetes, and diabetic complications.

Obesity becomes a major health problem in the world and is well associated with T2DM. Body mass index (BMI) is the most common method used in obesity. Waist circumference (WC) or the waist-to-hip ratio are used as surrogate indicators of body fat distribution. However, they are still not enough to assess accurately the body fat distribution (21). The previous study has shown that Asian population has a considerably higher body fat percentage (BF) for the same BMI when compared with Caucasians (22). Asians have a higher prevalence of metabolic dysregulation than those reported in Caucasian groups with equivalent BMI or WC (21, 23).

The information about the relationship between iron status and body fat distribution in diabetic patients is quite little. Therefore, the aim of this study was to determine the association between biomarkers of iron status and body fat distribution in patients with T2DM.

## Materials and Methods

### Study Population

Between January 2019 and August 2021, patients with T2DM were recruited from the Department of Endocrinology at the First Affiliated Hospital of Wenzhou Medical University. This study was a cross-sectional study. T2DM was diagnosed using 75 *g* oral glucose tolerance tests in accordance with the American Diabetes Association’s criteria. The inclusion criteria were age ≧ 18 years and diagnosis of T2DM. The exclusion criteria included tumors, blood system disease, hepatic failure, chronic renal impairment, inflammatory diseases, and recent iron supplementation or iron chelators therapy. All participants in our study signed the written informed consent. Finally, 824 participants aged between 19 and 81 years were recruited for this study.

### Data Collection

A detailed clinical history, such as age, gender, diabetes duration, hypertension, and medication usage, was obtained from participants’ medical records and through self-reporting. The BMI was calculated using the formula: BMI = weight (kg)/height (m^2^). Blood samples were collected from the antecubital vein in the overnight fasting state. Blood samples were used for estimating renal and liver functions, plasma glucose (by using standard enzymatic methods), glycated hemoglobin (HbA1c, by using high-performance liquid chromatography), and lipid profiles (by using standard enzymatic methods), such as total cholesterol (TC), high-density lipoprotein cholesterol (HDL-C), low-density lipoprotein cholesterol (LDL-C), and triglycerides (TG).

Serum ferritin levels were measured by chemiluminescence immunoassay (Beckman Coulter, Miami, FL, United States). Serum iron levels were measured by a ferrozine colorimetric method (serum iron detection kit, Beckman Coulter, Miami, FL, United States), complexed with the ferrozine iron reagent, at 562 nm at a fixed-time interval. The concentration of iron in the sample is directly proportional to the change in absorbance. and serum unsaturated transferrin binding capacity (UIBC) levels were measured by using a 2-nitroso-5-[N-n-propyl-N-(3-sulfopropyl) amino] phenol (nitroso-PSAP) assay (Beckman Coulter, Miami, FL, United States). Total iron-binding capacity (TIBC) was calculated as the sum of serum iron and UIBC, and the percentage of transferrin saturation (TSAT) as serum iron/TIBC × 100.

### Measurement of Body Fat

Body fat was measured by dual-energy X-ray absorptiometry (DXA; GE Healthcare, Madison, WI, United States) using Encore software. Total body weight was divided into bone mineral content, lean mass, and total fat mass. We calculated BF, android fat/total BF, gynoid fat/total BF, and visceral fat mass (VF). In addition, we analyzed the fat mass of the android body region and gynoid body region. Android-to-gynoid fat ratio (A/G ratio) was calculated as android fat mass divided by gynoid fat mass. The android region was defined as above the line between the iliac crests and below the horizontal line 20% of the distance between the inferior boundary and the chin. The gynoid region was defined as the upper boundary at 1.5 times the height of the android region below the iliac crests and the lower boundary at a line 2 times the height of the android region below the upper boundary.

### Statistical Analysis

The SPSS 23.0 software (SPSS Inc., Chicago, IL, United States) was used for statistical analysis. Data were presented as mean ± SD, percentages, or median values with an interquartile range. Differences between groups were tested using ANOVA. Count data were tested using χ^2^ tests. The linear regression models and logistic regression models were performed to determine the relationship between iron metabolism biomarkers and body fat composition, and odds ratios (ORs) between the comparison groups were obtained with a 95% confidence interval (CI). To assess the optimal cut points of iron metabolism biomarkers, a receiver operating characteristic (ROC) curve analysis was conducted. All tests were two sided, and *p* < 0.05 was considered statistically significant.

## Results

A total of 824 patients (486 men and 338 women, mean age 56.19 ± 12.35 years) were recruited for our study. Table 1 shows the baseline characteristics of the patients. Based on sex-specific tertiles of serum ferritin levels, participants were categorized into three subgroups. No significant differences in gender, age, BMI, HbA1c, and diabetes duration were found among the three groups (all *p* > 0.05). No significant differences in the levels of TC and HDL-C were observed among the three groups (all *p* > 0.05). WC, TG, LDL-C, and hypertension were all increased significantly with increased serum ferritin (all *p* < 0.05). Serum iron and TSAT were increased significantly with increased serum ferritin (all *p* < 0.05). TIBC was decreased significantly with increasing serum ferritin (*p* < 0.05). No significant difference in BF and gynoid fat/total fat mass was observed among the three groups (all *p* > 0.05). VF, android fat/total body fat mass, A/G ratio, and high-sensitivity C-reactive protein (hs-CRP) were all increased significantly with increased serum ferritin (all *p* < 0.05).

**TABLE 1:** Baseline characteristics of study participants according to sex-specific tertiles of ferritin levels.

	Tertiles of ferritin levels (ng/ml)			*P*-value
	T1 (low)	T2 (middle)	T3 (high)	
	Men:Ferritin ≦ 127.9	Men:127.9 < Ferritin ≦ 253	Men:Ferritin > 253	
	Women:Ferritin ≦ 93.2	Women:93.2 < Ferritin ≦ 207	Women:Ferritin > 207	
*n*	272	284	268	
Women, *n* (%)	104	124	110	0.42
Age (years)	56.95 ± 12.45	56.24 ± 12.22	55.35 ± 12.38	0.32
Diabetes duration (years)	8.95 ± 7.07	9.14 ± 7.07	10.08 ± 7.66	0.18
BMI (kg/m^2^)	23.81 ± 3.47	24.22 ± 3.52	24.00 ± 3.63	0.39
Waist circumference (cm)	87.2 ± 10.4	90.7* ± 10.9	92.5* ± 10.7	0.03
Hypertension (yes/no)	84/188	119/165	164/104*^#^	<0.001
HbA1c (%)	9.55 ± 2.29	9.89 ± 2.07	9.73 ± 2.30	0.21
TC (mmol/L)	4.99 ± 1.06	4.85 ± 1.38	4.81 ± 1.03	0.16
TG (mmol/L)	1.41 ± 0.47	1.77 ± 0.86*	2.59 ± 1.41*^#^	<0.001
HDL-C (mmol/L)	1.10 ± 0.78	1.05 ± 0.19	1.03 ± 0.23	0.20
LDL-C (mmol/L)	2.40 ± 0.83	2.62 ± 0.88*	2.79 ± 0.78*^#^	<0.001
Serum Iron (μmol/L)	14.28 ± 3.41	15.19 ± 4.01*	18.05 ± 5.47*^#^	<0.001
Ferritin (ng/ml)	78.66 ± 31.05	182.90 ± 38.05*	388.89 ± 196.49*^#^	<0.001
TSAT (%)	25.66 ± 20.96	30.68 ± 10.50*	33.33 ± 11.62*^#^	<0.001
TIBC (μmol/L)	52.53 ± 9.53	50.27 ± 28.30*	46.66 ± 8.32*^#^	0.001
BF (%)	26.36 ± 9.53	27.33 ± 18.58	27.66 ± 7.04	0.47
VF (kg)	0.97 ± 0.15	1.05 ± 0.12*	1.14 ± 0.12*^#^	<0.001
Android fat/total fat mass (%)	31.87 ± 11.48	32.37 ± 11.10	34.27 ± 11.24*^#^	0.03
Gynoid fat/total fat mass (%)	28.90 ± 9.25	27.36 ± 20.68	27.37 ± 8.73	0.35
A/G ratio	1.09 ± 0.24	1.22 ± 0.30*	1.26 ± 0.27*	<0.001
Hs-CRP	1.65 ± 0.60	1.70 ± 0.82*	2.30 ± 1.07*^#^	<0.001

*Indicates to p < 0.05 when compared with the T1 group.

*^#^Indicates to p < 0.05 when compared with the T2 group.*

Baseline demographic and clinical characteristics for the patient groups divided according to the A/G ratio are presented in Table 2. Patients with a high A/G ratio had a significantly higher WC, BF, android fat/total fat mass, VF, and A/G ratio. There were no significant differences in BMI and gynoid fat/total fat mass between the two groups. Patients with a high A/G ratio were more likely to have a higher prevalence of hypertension and had significantly higher TC, TG, LDL-C, and hs-CRP but significantly lower HDL. There were no significant differences in age, gender, diabetes duration, and HbA1c between the two groups. Patients with a high A/G ratio had significantly higher serum iron, ferritin, and TSAT but significantly lower TIBC.

**TABLE 2:** Baseline characteristics of study participants according to A/G ratio.

	A/G ratio		*P* value
	<1	≧1	
*N*	185	639	
**Body composition**			
BMI	23.82 ± 3.85	24.07 ± 3.45	0.42
Waist circumference	87.92 ± 10.83	91.75 ± 11.36	0.04
BF (%)	21.98 ± 8.18	28.61 ± 13.55	<0.001
VF (kg)	1.01 ± 0.15	1.07 ± 0.14	<0.001
Androin fat/total fat mass (%)	21.69 ± 10.55	36.05 ± 9.29	<0.001
Gynoid fat/total fat mass (%)	26.23 ± 15.89	28.34 ± 8.02	0.07
**Clinical parameters**			
Women, *n* (%)	78	260	0.72
Age (years)	56.07 ± 11.77	56.33 ± 12.91	0.80
Diabetes duration (years)	8.46 ± 6.19	9.65 ± 7.54	0.05
Hypertension (yes/no)	70	297	0.04
HbA1c (%)	9.89 ± 1.80	9.68 ± 2.33	0.25
TC (mmol/L)	4.36 ± 0.99	5.03 ± 1.18	<0.001
TG (mmol/L)	1.66 ± 0.68	2.00 ± 1.19	<0.001
HDL-C (mmol/L)	1.15 ± 0.27	1.04 ± 0.52	0.01
LDL-C (mmol/L)	2.43 ± 0.89	2.65 ± 0.83	0.002
Serum Iron (μmol/L)	14.98 ± 3.85	16.48 ± 5.12	0.001
Ferritin (ng/ml)	175.36 ± 98.15	227.11 ± 186.85	<0.001
TSAT (%)	27.04 ± 9.69	30.71 ± 16.57	0.004
TIBC (μmol/L)	53.68 ± 7.18	48.81 ± 20.26	0.001
Hs-CRP	1.45 ± 0.90	2.00 ± 0.86	<0.001

In the model adjusted for age and gender, a higher ferritin was associated with a higher VF in Table 3.

**TABLE 3:** Iron metabolism biomarkers and visceral fat mass.

	Visceral fat mass (kg) β (95%CI)		
	Module1	Module2	Module3
Serum iron	−0.01 (−0.04,0.01)	−0.01 (−0.04,0.01)	−0.02 (−0.06,0.01)
Ferritin	0.43^#^ (0.18,1.20)	0.44^#^ (0.19,1.23)	0.39^#^ (0.15,1.18)
TIBC	−0.04 (−0.08,0.02)	−0.04 (−0.08,0.02)	−0.01 (−0.05,0.02)
TSAT	0.03 (0.01,0.05)	0.02 (0.00,0.05)	0.01 (−0.02,0.03)
*R* ^2^	0.20	0.25	0.44

*Model 1: Unadjusted. Model 2: Model 1 additionally adjusted for age and gender. Model 3: Model 2 additionally adjusted for BMI, hs-CRP, total body fat, and waist circumference.*

*^#^Indicates to p < 0.01.*

**FIGURE 1:**
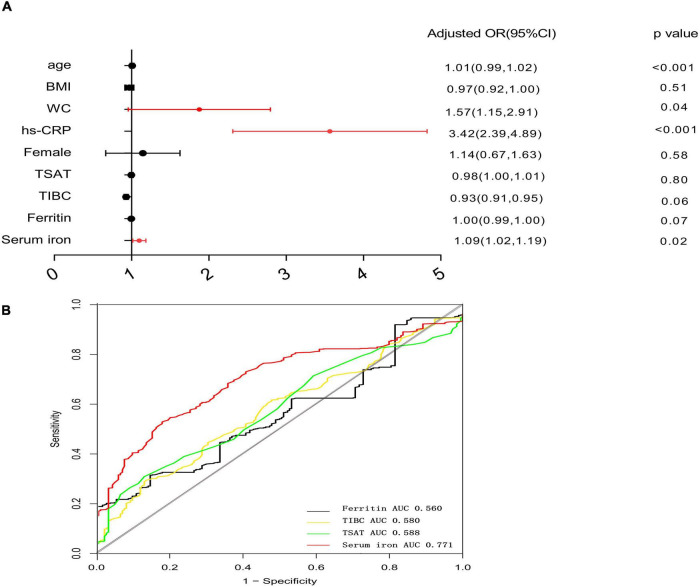
Forest plots for multivariate logistic analysis of variables for high A/G ratio and receiver-operating characteristic curve analysis of iron metabolism biomarkers for high A/G ratio. **(A)** Multivariate analysis: adjusted OR after adjustment for age, gender, BMI, WC, and high-sensitivity C-reactive protein (hs-CRP). **(B)** ROC analysis of iron metabolism biomarkers for a high A/G ratio. The cutoff value of serum iron for high A/G ratio was defined as the optimal sensitivity and specificity of the ROC curve. OR, odds ratio; ROC, receiver operating characteristic analysis; and A/G ratio, android-to-gynoid fat ratio.

The multivariate logistics analysis after adjustment for gender, age, BMI, WC, and hs-CRP, serum iron level is still significantly associated with a high A/G ratio (OR = 1.09, 95% CI, 1.02–1.19, *p* = 0.008) in Figure 1A. Figure 1B summarizes the cutoff value of iron metabolism biomarkers for the A/G ratio using a ROC curve analysis. The area under ROC (AUC) value of serum iron was 0.771 (95% CI: 0.649–0.893, *p* = 0.01). The cutoff value of serum iron for A/G ratio was 18.56 with a sensitivity of 88.9% and a specificity of 63.9%. The area under the ROC values of ferritin, TSAT, and TIBC were 0.560, 0.588, and 0.580, respectively.

## Discussion

In our study, it was observed that the patients with higher ferritin levels were suffered from more metabolism disorders that included dyslipidemia and hypertension. Several previous studies have indicated the similar results that increased ferritin levels are associated with disrupted lipid metabolism in T2DM (24–26). Higher serum iron levels, TSAT levels, and hs-CRP levels are found in the patients with the highest tertile of ferritin levels. There are some studies about the relationships between iron status and T2DM or diabetic complications. A Mendelian randomization study by Wang et al. has shown that genetically instrumented serum iron (OR: 1.07; 95% CI, 1.02–1.12), ferritin (OR: 1.19; 95% CI, 1.08–1.32), and TSAT (OR: 1.06; 95% CI, 1.02–1.09) are positively associated with T2DM. In contrast, genetically instrumented transferrin, a marker of reduced iron status, was inversely associated with T2DM (OR: 0.91; 95% CI, 0.87–0.96; 27). Kim et al. have reported that high dietary iron intake is related to the incidence of diabetic peripheral neuropathy (28). The potential pathways may be iron overload that induces oxidative stress and triggers insulin resistance (29, 30). Some recent studies found that iron released from ferritin is regulated by a process known as ferritinophagy, where the nuclear receptor coactivator 4 (NCOA4) directly binds the ferritin light chain and transfers the complex to the autolysosome for degradation (31). During the process, iron is released and the amount of free ferrous iron is increased, it is believed to lead to the generation of oxidative stress through Fenton and Haber-Weiss chemistry. Furthermore, in the pathological states, when the iron-binding capacity of transferrin is exceeded, non-transferrin bound iron can occur, which may induce toxic consequences (32).

To the best of our knowledge, there are a few studies about the relationship between iron status and body composition in T2DM. One of the most recent developments for whole-body DXA is the possibility to evaluate visceral adipose tissue. The clinical importance of visceral adipose tissue is widely recognized as a better predictor of mortality and an independent risk factor for the assessment of cardiovascular disease (CVD) risk (33, 34). Compared with DXA anthropometry, conventional anthropometry, such as BMI, waist, and hip circumference, underestimates the associations of obesity with T2DM and CVD risk markers. Patients with normal values of BMI may have an increased visceral adipose tissue, thus being at higher T2DM and CVD risk than that estimated by only conventional measurements (35). Our results reveal that higher VF is found in the patients with the highest tertile of ferritin levels. Furthermore, our study indicates that a higher serum ferritin level is positively associated with a higher VF.

Another parameter that can be used for evaluating the CVD risk is the A/G ratio, which is analog to the more commonly used anthropomorphic measurement of waist-to-hip ratio (36). Studies investigating body composition have reported that an increased android fat distribution (with values of AG ratio greater than 1) is associated with conditions, such as dyslipidemia, insulin resistance, CVD, and stroke (36–38). In the present study, our observations indicate that only a higher serum iron level has a significant relationship with the higher A/G ratio (A/G ratio ≧ 1). According to ROC analysis, the result indicates that the serum iron levels were significantly useful for making a clinical prediction for the presence of a high A/G ratio.

However, our results are different from the DIabetes CArdiovascular RIsk of VAllecas study by Vaquero et al. (39). The study has reported that low TSAT is highly prevalent in diabetes with obesity. A low TSAT value is a common indicator of absolute or functional iron deficiency. Additionally, high ferritin levels can falsely reflect iron status when patients are with inflammatory conditions. In contrast, lesser degrees of iron overload are required to raise TSAT levels. This in general makes increased TSAT a more sensitive marker than ferritin (40). Moreover, in the current article, we review the existing literature about the relationship between iron status and obesity without diabetes. An analysis based on a subsample from the cross-sectional Irish National Adult Nutrition Survey (2008–2010) by Heslin et al. has indicated that excessive body fat was significantly associated with increased serum hepcidin and ferritin and an increased prevalence of severe risk of iron overload (41). Hepcidin inhibits dietary absorption in the duodenum, the release of recycled iron from macrophages, and the exit of stored iron from hepatocytes by downregulation of ferroportin (42). Hepcidin is known to be induced by iron overload and inflammation. Therefore, more hepcidin is produced by hepatocytes when iron is excess (43). Furthermore, adipose tissue, particularly visceral, expresses and secretes other cytokines, such as inflammatory cytokine interleukin-6 (IL-6; 44). IL-6 can also trigger hepcidin induction *via* the IL-6R/STAT3 pathway (42). As a result, increased hepcidin induces cellular retention of iron, reducing iron export from enterocytes, macrophages, and hepatocytes, resulting in tissue iron overload. As a result, there is an increase in intracellular iron levels and oxidative stress (45). McClain et al. further demonstrated that adipocyte iron levels negatively regulate the transcription of adiponectin (46). Carlos López-Otín et al. have found a significant decrease in body fat, improved glucose tolerance and insulin sensitivity through blocking hepcidin expression in mice (47). Our study indicates that the serum iron levels and TSAT levels are significantly higher in the patients with a higher VF and a higher A/G ratio. Lobo et al. have found that liver iron accumulation occurred in the high-fat-diet-fed animals, and body adiposity was positively associated with liver iron stores but negatively associated with serum iron levels (48). Heslin et al. have revealed that the disturbances in iron homeostasis observed in individuals with excess adiposity are likely due to the inflammation of adipose-derived cytokines (41). Those at severe risk of iron overload display an increased prevalence of adipose tissue dysfunction. A recent study has shown that the adipose responds to iron overload at an early stage to interfere with lipid metabolism by secreting adipocytokines, which may further affect glucose metabolism, inflammation, and other iron overload-induced effects on the body (49). Therefore, there are inevitably interactions and influences among obesity, inflammation, and iron overload. In our study, we also report that hs-CRP is associated with ferritin and A/G ratio. More studies on specific mechanisms are needed to be confirmed by further investigations.

Some limitations exist in the present study. First, this study is a single-center cross-sectional study. Second, the biomarkers of iron status are affected by various factors. Iron intake data are not analyzed. Further long-term prospective cohort or interventional studies are needed to confirm the causality.

In conclusion, our data suggest that higher serum iron and ferritin concentrations were associated with a higher VF. Higher serum iron concentrations were positively correlated with a high A/G ratio. The relationship between iron overload and the change in body composition of T2DM may be bidirectional, with iron overload both cause and result of the adipose tissue dysregulation. However, future studies with a large number of subjects are needed to confirm these findings.

## Standard Biosecurity and Institutional Safety Procedures

All the biosafety measurements have been adopted and the institutional safety procedures are adhered. The laboratory of our institution has a biosafety level 2 (BSL-2) standard where all standards and protocols are adopted as per the guidelines of the Clinical and Laboratory Standards Institute (CLSI).

## Data Availability Statement

The original contributions presented in this study are included in the article/supplementary material, further inquiries can be directed to the corresponding authors.

## Ethics Statement

The studies involving human participants were reviewed and approved by the Ethics Committee of the First Affiliated Hospital of Wenzhou Medical College. The patients/participants provided their written informed consent to participate in this study.

## Author contributions

GX contributed to the conception and design of the study. ZF and ZP organized the database. LD performed the statistical analysis. CS wrote the first draft of the manuscript. CZ and CR wrote sections of the manuscript. All authors contributed to manuscript revision, read, and approved the submitted version.

## Conflict of Interest

The authors declare that the research was conducted in the absence of any commercial or financial relationships that could be construed as a potential conflict of interest.

## Publisher’s Note

All claims expressed in this article are solely those of the authors and do not necessarily represent those of their affiliated organizations, or those of the publisher, the editors and the reviewers. Any product that may be evaluated in this article, or claim that may be made by its manufacturer, is not guaranteed or endorsed by the publisher.
